# Assessing Clinicians’ Legal Concerns and the Need for a Regulatory Framework for AI in Healthcare: A Mixed-Methods Study

**DOI:** 10.3390/healthcare13131487

**Published:** 2025-06-21

**Authors:** Abdullah Alanazi

**Affiliations:** 1 Health Informatics Department, King Saud Ibn Abdulaziz University for Health Sciences, Riyadh 11481, Saudi Arabia; anaziabdul@ksau-hs.edu.sa or abdullahgcc@gmail.com; Tel.: +966-1419-5453; 2 King Abdullah International Medical Research Center, Riyadh 14611, Saudi Arabia

**Keywords:** artificial intelligence (AI), legal, clinicians, Saudi Arabia

## Abstract

**Background:** The rapid integration of artificial intelligence (AI) technologies into healthcare systems presents new opportunities and challenges, particularly regarding legal and ethical implications. In Saudi Arabia, the lack of legal awareness could hinder safe implementation of AI tools. **Methods:** A sequential explanatory mixed-methods design was employed. In Phase One, a structured electronic survey was administered to 357 clinicians across public and private healthcare institutions in Saudi Arabia, assessing legal awareness, liability concerns, data privacy, and trust in AI. In Phase Two, a qualitative expert panel involving health law specialists, digital health advisors, and clinicians was conducted to interpret survey findings and identify key regulatory needs. **Results:** Only 7% of clinicians reported high familiarity with AI legal implications, and 89% had no formal legal training. Confidence in AI compliance with data laws was low (mean score: 1.40/3). Statistically significant associations were found between professional role and legal familiarity (χ^2^ = 18.6, *p* < 0.01), and between legal training and confidence in AI compliance (t ≈ 6.1, *p* < 0.001). Qualitative findings highlighted six core legal barriers including lack of training, unclear liability, and gaps in regulatory alignment with national laws like the Personal Data Protection Law (PDPL). **Conclusions:** The study highlights a major gap in legal readiness among Saudi clinicians, which affects patient safety, liability, and trust in AI. Although clinicians are open to using AI, unclear regulations pose barriers to safe adoption. Experts call for national legal standards, mandatory training, and informed consent protocols. A clear legal framework and clinician education are crucial for the ethical and effective use of AI in healthcare.

## 1. Introduction

Artificial intelligence (AI) has developed as a transformative power in healthcare, designed to closely simulate the human intelligence in clinical intellectual and decision-making processes [1]. Current advancements, mostly in machine learning and large language models (LLMs), have simplified the comprehensive integration of AI tools into clinical workflows [2]. LLMs, for instance, can support clinical documentation and medical reasoning with more reliability, but so far, they have also advanced considerable legal and ethical concerns [3,4]. Remarkably, AI developers and vendors currently allow limited or no legal concern for protecting user data confidentiality or the outputs generated by these systems [5].

The urgency of regulatory misunderstanding was highlighted in a widely publicized case in 2024, when a 14-year-old boy died by suicide after prolonged interactions with an AI chatbot. This event flashed global argument over the ethical responsibility of AI developers, particularly in sensitive domains such as mental health care [6]. Such events highlight the hazards of deploying AI without clear legal precautions.

The legal concerns are mainly critical in clinical settings, where the hazards of misdiagnosis, inappropriate recommendations, or data misuse can directly affect patient safety [7]. One commonly mentioned example is IBM Watson for Oncology, primarily endorsed as an innovation tool for cancer treatment planning. However, its recommendations were frequently or dangerously inappropriate, leading to significant inadequacies in real-world validation and oversight [8,9]. Correspondingly, in 2023, UnitedHealth faced a class-action litigation after an AI-driven claims processing system left patients without medically necessary treatments. The algorithm’s use, despite its cost-saving aims, reportedly caused harmful delays in care and raised serious moral questions about algorithmic bias and corporate responsibility [10].

In contrast, successful AI applications, such as Google Health’s system used for detecting diabetic retinopathy, indicate the technology’s potential when ethical standards and validation rules are strong [11]. Until now, even this model, which excelled in controlled situations, wavered under real-world situations, predominantly in low-resource situations, due to such variables as image quality and lighting [12]. These results highlight a persistent challenge: the gap between AI’s practical performance in advanced stages and its consistency in miscellaneous clinical settings.

Such conflicting cases reflect the dual-edged nature of AI in healthcare. On one hand, AI can advance diagnostic accuracy, update workflows, and expand access to preventive facilities. On the other hand, lacking suitable legal, ethical, and technical precautions, it may aggravate inequities, affect patient safety, and diminish trust in healthcare systems [13,14].

Saudi Arabia has made significant developments in launching a governance environment for AI through national strategies and institutional contexts. The Saudi Data and Artificial Intelligence Authority (SDAIA), established in 2019, primes the application of the National Strategy for Data and Artificial Intelligence (NSDAI), the purpose of which is to position the kingdom as a worldwide AI leader by 2030 [15]. The NSDAI outlines goals about responsible AI development, ethical procedures, data authority, and capacity-building. In the healthcare field, the Saudi Food and Drug Authority (SFDA) has started the regulation of AI-powered medical technologies, together with software as a medical device (SaMD). The SFDA requires AI-based tools to meet classification standards, endure performance validation, and preserve post-market security monitoring [16]. In 2022, the SDAIA also issued the AI Ethics Principles, a national guideline highlighting human-centricity, equality, confidentiality, reliability, transparency, and responsibility in AI deployment [17]. Despite these enterprises, a keen legal framework specific to the clinical use of AI—addressing legal responsibility, transparency in decision-making, informed consent, and healthcare data integration across systems—is still deficient. This gap represents legal doubts and ethical hazards as Saudi Arabia accelerates digital health transformation under Vision 2030 and the Health Sector Transformation Program [18].

This study’s objective is to explore clinicians’ perceived legal concerns relating to the use of AI in healthcare inside the Saudi context. It further aims to offer legal recommendations for evolving an initial legal framework that supports the safe, curated, ethical, and effective integration of AI technologies into clinical and medical practice.

## 2. Materials and Methods

Study Design

This study employed a sequential explanatory mixed-methods design to explore clinicians’ legal concerns regarding the use of artificial intelligence (AI) in healthcare and to assess the perceived need for a dedicated regulatory framework. The mixed-methods approach allowed for quantitative measurement of awareness, perceptions, and training needs, followed by qualitative inquiry to contextualize the findings and identify policy-relevant themes.

The study consisted of two phases:

- Phase One: A structured quantitative survey distributed among clinicians to assess awareness, training, liability concerns, and trust in AI technology.
- Phase Two: An expert panel session involving legal, clinical, and policy professionals to explore barriers, ethical considerations, and regulatory priorities in greater depth.

This design was chosen to ensure both breadth (via survey data) and depth (via expert interpretation) in addressing the study objectives.

Phase One: Quantitative Component

Target Population and Sampling

The survey targeted healthcare professionals including physicians, nurses, and allied health practitioners across public and private institutions in Saudi Arabia, see Appendix A. A convenience sampling approach was used, leveraging institutional mailing lists and professional networks to distribute the questionnaire. To ensure representation across specialties, an open invitation was extended through regional medical societies and hospital departments.

A total of 357 respondents completed the survey during the data collection window (March, April 2025).

Survey Instrument

A self-administered structured questionnaire titled “Assessing Clinicians’ Legal Concerns and the Need for a Regulatory Framework for AI in Healthcare” was developed based on a review of the literature and expert input. The tool comprised 21 close-ended items grouped into six domains:

1. Legal Awareness;
2. Liability Concerns;
3. Data Privacy and Security;
4. Ethical and Legal Framework;
5. Trust in AI Technology;
6. Future Regulatory Directions.

In addition, demographic data such as professional role, years of experience, and region of practice were collected. One open-ended field invited further comments.

The survey was pilot tested with 15 clinicians to ensure clarity and relevance. Feedback informed minor linguistic adjustments. The internal consistency was calculated and revealed an acceptable level (>0.7).

Data Collection and Analysis

The survey was distributed electronically (emails with hyperlink) via Google Forms. Participation was voluntary, and anonymity was maintained throughout.

Descriptive statistics (frequencies, percentages, and means) were used to summarize the data. Inferential analyses were conducted using SPSS Version 27:

- Chi-square tests were used to examine associations between categorical variables (e.g., role and legal familiarity).
- Independent-sample *t*-tests were used to compare confidence levels between trained and untrained participants.
- Pearson correlation analysis assessed the relationship between legal familiarity and willingness to use AI tools lacking legal clarity. A *p*-value < 0.05 was considered statistically significant.

Phase Two: Qualitative Component

Expert Panel Design

Following the survey, a qualitative expert panel was convened to interpret findings and identify legal and regulatory challenges specific to the Saudi context. The panel consisted of 12 experts, including the following:

- Health law specialists;
- Medical ethics faculty;
- Senior clinicians from AI-use specialties;
- Digital health policy advisors.

Selection was purposive, based on expertise in AI regulation, clinical governance, and health informatics.

Discussion Guide:

The session was guided by ten open-ended questions designed to elicit discussion around the following (see Appendix B):

- Legal accountability;
- Data privacy laws (e.g., PDPL, GDPR compliance);
- Liability frameworks;
- Regulatory oversight and transparency;
- Barriers to AI implementation in the Saudi healthcare system.

The questions were informed by preliminary survey findings and reviewed by two scholars for content validity.

Data Collection and Analysis

The panel was conducted via a moderated Zoom session and recorded with participant consent. Notes and transcripts were subjected to thematic analysis using Braun and Clarke’s six-step framework. Codes were generated inductively, and themes were cross-validated by two independent coders.

Emerging themes were triangulated with the survey findings to build a comprehensive picture of the legal, ethical, and institutional concerns surrounding AI adoption in healthcare.

## 3. Results

Phase One—Quantitative Analysis

This phase of the study aimed to assess participants’ familiarity with the legal implications of AI, their confidence in AI systems’ compliance with data laws, and the extent of formal legal training received in the context of AI. A total of 357 healthcare professionals across Saudi Arabia including physicians (40%), nurses (33%), and allied health practitioners (27%) from both public and private sectors participated. Participants represented diverse regions and specialties, with an average of 6.7 to 11.2 years of experience across roles. See Table 1.

Clinicians’ Familiarity, Confidence, and Legal Training Regarding AI Legal Aspects

The study revealed a significant lack of legal awareness among healthcare professionals regarding the use of artificial intelligence, as indicated by Table 2. When asked about their familiarity with the legal implications of AI, a majority of participants (58.5%, n = 209) reported being “Not Familiar at All,” while 34.5% (n = 123) indicated they were “Somewhat Familiar”. Only a small fraction (7.0%, n = 25) considered themselves “Very Familiar”. The mean familiarity score was 1.49 (on a 3-point scale), reflecting generally limited understanding of the legal responsibilities and risks associated with AI integration in healthcare settings.

A similar pattern emerged in participants’ confidence in AI systems’ compliance with data protection and privacy laws. Most respondents (64.7%, n = 231) indicated they were “Not Confident”, while 30.3% (n = 108) were “Somewhat Confident”, and only 5.0% (n = 18) expressed being “Very Confident”. The mean confidence score of 1.40 (on a 3-point scale) highlights a widespread skepticism about whether current AI technologies meet legal and ethical standards, particularly concerning patient data security and privacy.

Regarding formal training on the legal aspects of AI, an overwhelming majority of participants (89%, n = 318) reported they had not received any such training, while only 11% (n = 39) confirmed that they had. When treated as a binary variable (1 = Yes, 0 = No), the mean response was 0.11, underscoring a critical gap in professional development. This lack of structured legal education may contribute to the observed low levels of familiarity and confidence, suggesting a need for targeted training and policy interventions to better prepare clinicians for the legal challenges posed by AI in healthcare.

Inferential Statistical Analysis

To explore deeper relationships between key variables, a series of inferential statistical tests were conducted, as described by Table 3. These analyses were aimed at identifying associations between professional roles and familiarity with legal implications, evaluating the effect of formal legal training on confidence in AI compliance, and assessing the relationship between familiarity and willingness to adopt AI systems without legal safeguards. First, a chi-square test of independence examined how professional roles related to familiarity with AI’s legal implications. The results showed a statistically significant association (χ^2^ ≈ 18.6, *p* ≈ 0.001), with physicians reporting higher levels of legal familiarity compared to nurses and other healthcare professionals.

Next, an independent-sample *t*-test compared confidence in AI’s compliance with data laws between participants who had received formal legal training and those who had not. Those with legal training (n = 39) had a significantly higher mean confidence score (M = 1.92, SD = 0.58) than those without training (n = 318; M = 1.33, SD = 0.60), with a highly significant *t*-value (≈6.1, *p* < 0.001). This indicates that formal legal training substantially boosts confidence in AI’s legal compliance.

Finally, a Pearson correlation analysis was conducted to examine the relationship between familiarity with legal implications and willingness to use AI systems without explicit legal safeguards. The results revealed a moderate positive correlation (r ≈ 0.42, *p* < 0.01), indicating that individuals with higher legal awareness tend to be more open to adopting AI even in the absence of formal legal protections. However, it is important to interpret this correlation with caution, as it does not imply a causal relationship. Conclusions drawn from this association should be approached carefully. Overall, the findings suggest that professional role and legal education shape both legal familiarity and confidence, and that increased legal awareness is linked to greater willingness to adopt AI in legally uncertain contexts.

Phase Two: Qualitative Study

Expert Insights on Legal Barriers to AI Adoption in Saudi Healthcare

To complement the quantitative findings, a qualitative analysis was conducted through a thematic synthesis of expert discussions, exploring legal and ethical impediments to the clinical adoption of AI in Saudi Arabia, as described by Table 4. The discussions were contextualized within the framework of Saudi Vision 2030 and evolving regulatory structures such as the Saudi Data and Artificial Intelligence Authority (SDAIA) and the Personal Data Protection Law (PDPL).

1. Legal Literacy is Critically Low

One of the most salient findings was the widespread lack of legal awareness among clinicians regarding AI use. Over 58.5% of surveyed healthcare professionals reported unfamiliarity with legal implications related to AI technologies. Experts interpreted this as a significant risk factor, particularly within institutions that operate under the evolving framework of the PDPL, which mandates strict controls on data usage, sharing, and international transfers—especially for sensitive health data. One participant mentioned that “I use AI tools occasionally, but I haven’t received any training or guidance about the legal implications. It’s a bit concerning, but no one has explained it to me”. Also, another participant said “There’s a lot of talk about AI improving healthcare, but I haven’t seen clear information on what’s legally permissible or what liabilities might exist”.

Barrier: A systemic absence of legal training or awareness programs targeting healthcare professionals has created a knowledge vacuum. Despite national AI ambitions, the front-line workforce remains unprepared for AI-related legal challenges.

2. Concerns About Data Protection and Privacy

An additional theme was the pervasive concern regarding data privacy and security. A notable 64.7% of clinicians expressed low confidence in AI systems’ compliance with Saudi data protection laws. Experts attributed this to a lack of transparency surrounding AI data practices, particularly with AI systems hosted on cloud platforms or built by international vendors.

Further compounding this issue is the absence of standardized consent protocols for AI-assisted care, leaving clinicians uncertain about their legal liability when deploying such technologies. One clinician mentioned that “While I see the potential of AI in improving patient care, I’m deeply concerned about how personal health data is being handled. Without clear assurances on data protection and privacy, it’s hard to fully trust these technologies—especially when sensitive information could be misused or exposed”.

Barrier: The ambiguity around compliance with the PDPL, combined with inadequate institutional guidance, undermines clinician trust in AI systems and increases legal exposure.

3. Training and Policy Integration Are Lacking

The study also highlighted a major gap in training: only 11% of participants had received any form of legal training related to AI. Experts criticized the Saudi Commission for Health Specialties (SCFHS) for not integrating AI legal risk management into Continuing Medical Education (CME) requirements.

Institutions similarly lack AI compliance infrastructures such as legal oversight units or designated AI officers, which leaves clinicians without formal support or policy backing when deploying AI tools.

Barrier: There is no institutional or regulatory mandate for AI-specific legal training, resulting in a workforce that is both uninformed and unsupported in managing AI risks.

4. Professional Role Influences Legal Awareness

Inferential statistical analysis confirmed that physicians possess greater legal awareness compared to nurses and technicians (χ^2^, *p* < 0.01). Experts observed that this disparity aligns with the hierarchical structure of Saudi healthcare, where physicians often bear ultimate medico-legal responsibility.

However, the use of AI systems typically involves interdisciplinary teams, and the lack of legal awareness among non-physicians creates asymmetric liability and operational risk, particularly in high-stakes environments like ICUs or telemedicine platforms.

Barrier: Legal literacy is unevenly distributed, making lower-tier staff vulnerable to uninformed use of AI and its legal consequences.

5. Legal Training Enhances Confidence in AI Use

Quantitative data revealed a statistically significant relationship between legal training and clinician confidence in AI compliance (*p* < 0.001). Qualitative discussions corroborated this, showing that clinicians with legal knowledge were more comfortable with integrating AI tools into their workflows.

Experts advocated for the development of national certification programs in AI governance, paralleling existing models for infection control or ACLS certification.

Barrier: Confidence is closely tied to exposure and training. Without formal education, clinicians remain skeptical and hesitant to use AI technologies.

6. Legal Familiarity Drives AI Adoption Willingness

A moderate positive correlation (r = 0.42, *p* < 0.01) was observed between legal familiarity and willingness to use AI. Clinicians expressed a clear desire to adopt AI tools but only under clear legal safeguards.

Expert commentary emphasized that even highly usable or accurate AI systems will face adoption barriers if clinicians fear legal repercussions. Thus, legal assurance is a more powerful driver of adoption than technical performance alone. One participant said “As someone who’s taken legal training, I feel much more comfortable integrating AI into our clinical workflows”.

Barrier: Legal ambiguity discourages innovation, as fear of liability outweighs the perceived benefits of AI integration.

7. Systemic Legal Barriers Specific to the Saudi Context

The expert panel identified a comprehensive set of legal barriers that are unique to the Saudi regulatory and institutional landscape:

## 4. Discussion

This study provides significant insights concerning the legal awareness and interest of clinicians in Saudi Arabia about the integration of artificial intelligence (AI) in healthcare services. As AI technologies gain traction in medical workflows, extending from diagnostics to decision support, consideration of their legal consequences has become significant for safeguarding patients, confirming ethical implementation, and sustaining professional responsibility. While our findings are in alignment with international patterns of limited legal familiarity and regulatory uncertainty, they also reveal dissimilar challenges related to Saudi Arabia’s growing legal landscape, cultural situation, and policy direction under Vision 2030.

One of the most obvious findings was the prevalent lack of legal literacy among clinicians. Almost 59% of participants stated no familiarity with the legal parts of AI use in clinical situations. This aligns with worldwide studies reporting similar gaps in legal consideration, mostly in relation to informed consent, data confidentiality, and responsibility when AI is used as a clinical support [19,20]. The clinicians often struggle with consideration of regulatory documents or understanding the differences between ethical and legal commitments in algorithmic decision-making [21,22]. In the Saudi framework, this issue is heightened by the quite recent rollout of the Personal Data Protection Law (PDPL) that, despite its importance, has not yet been fully integrated into established protocols or health education. This regulatory interval creates uncertainty around legal concern and increases the risk of inconsistent AI implementation.

The concerns about data confidentiality and compliance were also prominent. Almost two-thirds of clinicians expressed low confidence in AI systems’ adherence to data regulations. Internationally, such concerns are commonly linked to the blurred nature of algorithmic decision-making and the contribution of third-party, usually external, AI vendors [23]. The pressure between embracing advanced international AI solutions and maintaining national legal principles presents an important regulatory challenge that necessitates synchronization of both global technical regulations and local cultural frameworks [24,25].

The lack of well-structured legal training appeared as another contributing aspect to clinicians’ hesitation. Only 11% of respondents had acknowledged formal instruction on AI-related legal topics, a gap reflected in international studies show that medical training commonly prioritizes clinical and technical skills while overlooking governance and legal awareness [26,27]. The absence of regulatory commands from bodies like the Saudi Commission for Health Specialties (SCFHS) further exacerbates this gap, in spite of the rising clinical impact of AI in diagnostics, triage, and patient communication.

Role-based differences were also detected, with physicians bearing significantly greater legal familiarity than nurses and allied health professionals. This reflects present trends in the literature noting that legal responsibility tends to focus at senior decision-making levels [28]. Still, AI tools are progressively deployed in team-based care settings, raising concerns about common accountability. Authorized gaps among mid-level or junior healthcare specialists may contribute to varying or risky AI use, particularly in high-pressure environments like intensive care units or telemedicine.

Encouragingly, the study reported that legal training significantly increased clinician confidence in AI tools. Persons with prior experience with legal and regulatory content showed stronger trust in AI systems, a concept supported by international observations representing that even short-term interventions can improve professional willingness to adopt developing technologies responsibly [29]. The positive link between legal familiarity and willingness to use AI even in the absence of clear legal safeguards indicates that knowledge plays a critical enabling role in technology acceptance [30]. These remarks also highlight the boundaries of assuming that technological progress alone is enough for successful implementation; legal clarity and ethical guarantees are similarly critical.

Notably, the professional panel acknowledged a set of regulatory and institutional barriers specific to the Saudi context. These include unclear liability attribution, the lack of AI-specific informed consent forms, inadequate audit documentation, and institutional uncertainty about PDPL alignment. Although comparable structural gaps have been recognized globally, mainly around transparency and clearness in AI models [31,32], Saudi Arabia’s unique policy setting, cultural considerations, and reliance on international technology providers necessitate a tailored regulatory approach. Current work in Saudi legal literature highlights the urgent need for policy frameworks that echo both cultural values and modern legal tools, including digital authority and data ethics [33,34].

From an ethical perspective, the findings also raise concerns around equity and access. If authorized knowledge and regulatory assurance are focused among certain clinical groups (e.g., physicians), then implementation of AI possibly will inadvertently support existing orders, leaving nurses or technicians exposed to errors or legal consequences they are ill-prepared to deal with. Additionally, the absence of AI-specific informed consent limits patients’ autonomy, possibly violating ethical ethics that need transparency in decision-making tools that influence their care. These topics point to a rising need for shared accountability frameworks, where all members of a care team, not just senior clinicians, are mindful of and protected by the same legal standards.

In summary, this study suggests that while Saudi Arabia faces many of the same legal and ethical challenges recognized globally, i.e., low clinician legal knowledge, inadequate trust in compliance, and gaps in training, it must also confront regulatory issues related to its cultural and legal environment. Addressing these issues will necessitate synchronized action across ministries, professional regulators, and healthcare institutions to align AI deployment with national values and global best practices.

Recommendations

The findings of this study highlight the vital need for well-structured and informative transformations to bridge the legal literacy gap surrounding AI in Saudi healthcare. First and principally, a comprehensive national legal framework must be developed to direct the deployment and use of AI in clinical situations. This framework should be collaboratively produced by the Ministry of Health (MoH), the Saudi Data and Artificial Intelligence Authority (SDAIA), and the National Health Information Center (NHIC). It must encompass significant legal dimensions such as responsibility, informed consent, data protection, assessing, monitoring and compliance, all while remaining in concordance with the Personal Data Protection Law (PDPL) and ethical standards.

In parallel with regulatory modification, educational institutions and licensing bodies must prioritize legal literacy. The Saudi Commission for Health Specialties (SCFHS) should mandate legal training on AI ethics, data governance, and medico-legal responsibility as part of its continuing education necessities. Such training should not be limited to physicians but extended to nurses, allied health professionals, and technicians who regularly interact with AI tools in practice. Developing AI governance certification programs would further professionalize this domain and ensure a standardized baseline of knowledge across healthcare roles.

To improve transparency and patient autonomy, the development of AI-specific informed consent protocols is also essential. These consent forms should clearly explain the role of AI in clinical decision-making, the level of its autonomy, the possible risks involved, and how patient data is collected, managed, and protected. Adapting these forms to meet both international best practices and local cultural and legal norms, including agreement with PDPL and cultural values, would foster greater patient trust and institutional responsibility.

The hospitals and healthcare institutions should also take proactive steps by designating AI compliance officers. These would be responsible for managing AI integration, confirming tools meet regulatory standards, and organizing staff training and internal audits. In addition, AI tools should undergo formal pre-deployment evaluations to assess compliance with national legal frameworks and ethical guidelines, including cross-border data processing and data authority concerns.

Finally, the establishment of a general AI incident reporting system would provide a mechanism for clinicians and institutions to report legal or ethical concerns rising from AI use. This would permit regulatory bodies to monitor recurrent issues, assess the impact of interventions, and enhance legal frameworks in an approachable and data-driven style. Taken together, these recommendations aim to support the authorized and legally sound integration of AI in Saudi healthcare while strengthening clinician confidence and protecting patient rights.

Study Limitations

While the study offers significant insights into the legal alertness and attitudes of Saudi clinicians concerning AI use, some limitations should be recognized. The sample, though diverse in clinical roles, may not fully represent the entire healthcare segment in Saudi Arabia. Further, most respondents were associated with public hospitals, and perceptions from private sector experts, who may cooperate differently with global AI vendors or internal strategies, are underrepresented. Accordingly, the generalizability of the findings to the wide-ranging healthcare workforce may be restricted.

Moreover, the data are self-reported, which presents possible biases. Applicants may have over-estimated or under-estimated their familiarity with legal perceptions due to misunderstanding, recall bias, or the effect of social desirability. These individual insights, while respected, do not substitute for unbiased assessments of legal knowledge or competence.

The cross-sectional nature of the study presents another restriction. It captures clinicians’ views at a solitary point in time throughout a period of regulatory change as Saudi Arabia continues to implement the PDPL and other AI-related policies. Observations and levels of awareness may advance rapidly as new training programs, guidelines, or institutional strategies are presented. Longitudinal studies would be helpful in tracking how legal literacy and attitudes vary in response to such advances.

Another limitation is the study’s limited analysis of institutional variability. While clinicians from multiple hospitals participated, the study did not analytically examine how organizational features, such as hospital size, academic affiliation, or presence of legal sectors, influence AI governance practices and clinician readiness. This signifies an important avenue for more future research.

Lastly, the study focused entirely on clinical staff and did not include legal experts, hospital administrators, or policymakers, all of whom play important roles in shaping and enforcing AI-related guidelines. future research would benefit from a multi-stakeholder approach that incorporates perspectives from these essential actors to build a more holistic understanding of AI readiness and legal risk management in the healthcare system.

## 5. Conclusions

This study reveals major gaps in legal readiness among Saudi healthcare providers as AI becomes more integrated into clinical care. Many clinicians lacked familiarity with key laws like the PDPL and were uncertain about AI’s compliance with privacy standards. Concerns included unclear liability for AI errors, lack of informed consent procedures, and weak institutional oversight. These issues reflect global challenges but are intensified by local cultural and regulatory factors. Clinicians with legal training felt more confident and willing to adopt AI, showing the value of legal education. Legal knowledge varied across roles, suggesting training should include all healthcare staff. To safely advance AI under Vision 2030, Saudi Arabia must establish a national legal framework, integrate legal education into clinical practice, and strengthen institutional governance.

## Figures and Tables

**Table 1:** Demographic characteristics of healthcare professionals participating in the study.

Professions	Count (%)	Years of Experience (M, SD)	Specialization	Region
Physician	143 (40%)	11.2 ± 6.7	Internal Medicine, Surgery, Radiology	Riyadh, Jeddah, Dammam
Nurse	119 (33.3%)	8.4 ± 5.2	General Nursing, ICU, ER	Riyadh, Medina, Eastern Region
Allied Health	95 (26.6%)	6.7 ± 4.8	Radiology Tech, Pharmacy, Lab	Abha, Tabuk, Qassim

**Table 2:** Clinicians’ familiarity, confidence, and legal training regarding AI legal aspects.

Variable	Response Category	Frequency (%)	Mean (Scale)
Familiarity with Legal Implications	Very Familiar	25 (7.0%)	1.49 (1–3)
	Somewhat Familiar	123 (34.5%)	
	Not Familiar at All	209 (58.5%)	
Confidence in AI Legal Compliance	Very Confident	18 (5.0%)	1.40 (1–3)
	Somewhat Confident	108 (30.3%)	
	Not Confident	231 (64.7%)	
Received Formal Legal Training	Yes	39 (11%)	1.49 (1–3)
	No	318 (89%)	

**Table 3:** Inferential analysis of factors influencing legal awareness and confidence in AI use.

Statistical Test	Variables Compared	Key Results	Interpretation
Chi-Square Test	Role × Familiarity with AI Legal Aspects	χ^2^ ≈ 18.6, df = 4, *p* ≈ 0.001	Significant association: physicians more familiar than other roles
Independent T-Test	Legal Training × Confidence in AI Legal Compliance	t ≈ 6.1, *p* < 0.001	Clinicians with legal training showed significantly higher confidence
Pearson Correlation	Familiarity × Willingness to Use AI Without Legal Cover	r ≈ 0.42, *p* < 0.01	Moderate positive correlation: familiarity increases willingness to adopt AI

**Table 4:** Legal Barriers to AI Adoption in Saudi Healthcare

Barrier	Explanation
Ambiguous liability	No clear legal precedent identifies who is liable when AI fails—clinician, vendor, or hospital.
Lack of AI compliance documentation	Most hospitals lack legal documentation protocols, such as audit trails or checklists for AI deployment.
PDPL alignment uncertainty	Foreign AI systems may not comply with PDPL standards, particularly on data localization and cross-border sharing.
No AI-specific informed consent	Current consent forms do not disclose the use of AI or its decision-making role to patients.
Absence of training mandates	There is no requirement for legal or regulatory training on AI for clinicians.
Data sovereignty issues	Cross-border data flow involving AI systems may conflict with national digital sovereignty goals.

## Data Availability

The original contributions presented in this study are included in the article. Further inquiries can be directed to the corresponding author.

## Appendix A

Survey Instrument: Assessing Clinicians’ Legal Concerns and the Need for a Regulatory Framework for AI in Healthcare

Demographic Information

1. Role/Title: ___________________________________
2. Years of Experience: __________________________
3. Specialization: ________________________________
4. Location/Region: ______________________________
  Section 1: Awareness of Legal Implications
5. How familiar are you with the legal implications of using AI in healthcare?
  ☐ Very Familiar ☐ Somewhat Familiar ☐ Not Familiar at All
6. Do you believe AI in healthcare is subject to specific legal regulations in your country/region?
  ☐ Yes ☐ No ☐ Unsure
7. Have you received any formal training or education regarding the legal aspects of AI use in healthcare?
  ☐ Yes ☐ No
  Section 2: Liability Concerns
8. Who should be held legally accountable if an AI system in healthcare provides incorrect or harmful recommendations?
  ☐ Healthcare Providers (Clinicians)
  ☐ AI Developers or Manufacturers
  ☐ Hospital/Healthcare Institution
  ☐ No One (AI should be fully responsible)
  ☐ Other (Please specify): _________________________
9. How concerned are you about potential malpractice claims related to AI-driven decisions in patient care?
  ☐ Very Concerned ☐ Moderately Concerned ☐ Not Concerned
10. Would you use an AI system in clinical practice if it lacks clear liability clauses and legal protections?
  ☐ Yes ☐ No ☐ Unsure
  Section 3: Data Privacy and Security
11. Do you have concerns about the security and privacy of patient data when using AI technologies in healthcare?
  ☐ Yes ☐ No
12. How confident are you that AI systems comply with data protection laws (e.g., GDPR, HIPAA)?
  ☐ Very Confident ☐ Somewhat Confident ☐ Not Confident
13. Should patients be informed and consent to using their data to train AI models in healthcare?
  ☐ Yes ☐ No ☐ Unsure
  Section 4: Ethical and Legal Framework
14. Should there be a specific legal framework or set of regulations governing the use of AI in healthcare?
  ☐ Yes ☐ No ☐ Unsure
15. How comfortable are you with AI making critical decisions in patient care without direct human oversight?
  ☐ Very Comfortable ☐ Somewhat Comfortable ☐ Not Comfortable
16. Do you believe AI in healthcare could result in biased or discriminatory outcomes that may lead to legal challenges?
  ☐ Yes ☐ No ☐ Unsure
  Section 5: Trust in AI Technology
17. How much trust do you place in AI technologies to deliver accurate, unbiased healthcare recommendations?
  ☐ High Trust ☐ Moderate Trust ☐ Low Trust
18. Should the legal system step in to regulate AI in healthcare more strictly to prevent potential harm?
  ☐ Yes ☐ No ☐ Unsure
19. Would you feel more confident using AI in patient care if there was greater legal clarity regarding its role, use, and accountability?
  ☐ Yes ☐ No ☐ Unsure
  Section 6: Future Directions
20. In your opinion, what legal measures should be prioritized to ensure the safe and ethical use of AI in healthcare?
  ☐ Clear Liability Guidelines
  ☐ Strict Data Privacy and Security Regulations
  ☐ Ethical Standards for AI Use
  ☐ Transparent AI Algorithms
  ☐ Other (Please specify): _________________________
21. What further actions would you recommend to address the legal concerns surrounding AI in healthcare?
  ☐ Mandatory Legal Training for Healthcare Providers
  ☐ Government or Industry Regulations
  ☐ Legal Protections for Patients
  ☐ Increased Research and Advocacy
  ☐ Other (Please specify): _________________________

Additional Comments:

(Feel free to add any other comments or concerns related to the use of AI in healthcare).

## Appendix B

All Expert Panel Discussion Guide: Legal and Ethical Framework for AI in Healthcare.

The following questions were presented to a panel of experts to gather in-depth insights on legal challenges and regulatory frameworks for AI in healthcare.

1. What are the top legal barriers to implementing AI in healthcare?
2. How should healthcare organizations ensure AI systems comply with current regulations?
3. What legal measures are needed to protect patient privacy and data security in AI applications?
4. How should liability be handled if AI makes an error in patient care?
5. What role should regulatory bodies play in overseeing AI in healthcare?
6. What ethical principles should guide the creation of a legal framework for AI in healthcare?
7. How can a legal framework for AI balance innovation and patient safety?
8. What regulatory gaps need to be addressed as AI technology evolves in healthcare?
9. What collaborations are essential to develop a legal framework for AI in healthcare?
10. How can legal frameworks ensure AI systems are transparent and accountable in clinical decision-making?
